# Clinical features of patients with cerebral venous sinus thrombosis at plateau areas

**DOI:** 10.1002/brb3.2998

**Published:** 2023-04-24

**Authors:** Yongxiang Yang, Jingmin Cheng, Yuping Peng, Yan Luo, Dongbo Zou, Yongjian Yang, Yuan Ma

**Affiliations:** ^1^ Department of Neurosurgery The General Hospital of Western Theater Command Chengdu China; ^2^ Department of Oncology The General Hospital of Western Theater Command Chengdu China; ^3^ Department of Cardiology The General Hospital of Western Theater Command Chengdu China

**Keywords:** cerebral venous sinus thrombosis, coagulation function, plateau area, plain area, MRI

## Abstract

**Objective:**

Cerebral venous sinus thrombosis (CVST) is believed to be associated with high‐altitude exposure and has worse clinical prognosis in plateau areas than in plain areas, although this needs to be further verified. This retrospective study aims to compare the clinical differences of patients with CVST in plateau and plain areas and further ascertain the role of high‐altitude exposure in the etiology of aggravating predisposition toward CVST.

**Methods:**

Twenty‐four symptomatic CVST patients occurring at plateau areas (altitude ≥ 4000 m), in corresponding with 24 CVST patients occurring at plain areas (altitude ≤ 1000 m), were recruited according to the inclusion and exclusion criteria from June 2020 to December 2021. The collected data and compared parameters include clinical features, neuroimaging findings, hematology profile, lipid profile, and coagulation profile within 24 h of hospital admission, as well as the treatment method and final outcome.

**Results:**

There were no obvious differences of demographic characteristics, including gender, age, height, and weight between patients with CVST in plateau and plain areas, as well as medical history, neuroimaging findings, treatment protocols, and clinical outcome (all *p* > .05). Compared to patients with CVST at plain areas, time before hospital admission was longer and heartbeat was slower in patients with CVST at plateau areas (all *p* < .05). More importantly, elevated red blood cells counts, hemoglobin level, and altered coagulation function were found in patients with CVST at plateau areas (all *p* < .05).

**Conclusion:**

CVST patients in plateau areas presented with altered clinical characteristics, altered coagulation function, and aggravated predisposition toward venous thromboembolism compared with CVST patients in plain areas. Future prospective studies will be needed to further elucidate the influences of a high altitude on the pathogenesis of CVST.

## Graphical abstract

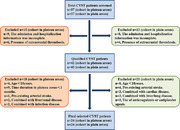

## 1 INTRODUCTION

Cerebral venous sinus thrombosis (CVST) is a rare form of neurovascular disorder, accounting for 0.5%–1% of all stroke patients and often affects the young population (Liang et al., 2017). The major pathological mechanism of CVST is the thrombosis of cerebral veins and/or dural sinus, which further leads to the reduction of CSF resorption and the elevation of intracranial pressure (Silvis et al., 2017). CVST has a variety of predisposing factors, age of onset, clinical manifestations, and radiological presentations. The most common risk factors of CVST include genetic/acquired prothrombotic conditions, infections, malignancy, pregnancy, oral contraceptives, and so on (Bano et al., 2021). In recent years, a relationship between high‐altitude exposure and CVST has been established, as a result of the documentation of CVST at high altitude has raised obviously owing to the availability of better neuroimaging facilities, such as MRI and MRV (Devasagayam et al., 2016; Khattar et al., 2019). The diagnosis of CVST occurring at high altitude is often delayed, as CVST is a rare disease, and its clinical symptoms and radiologic findings are usually similar with other neurological conditions occurring at high altitude such as altitudinal sickness (Hassan et al., 2019). As the timely diagnosis and treatment of CVST occurring at high altitude needs a more thorough understanding of the clinical presentation, it is essential to conduct research to clarify the clinical features of patients with CVST at plateau areas by comparing them with CVST patients at plain areas.

The clinical symptoms of CVST are variably ranging from isolated headache to focal neurological symptoms and signs, seizures, and coma (Gazioglu et al., 2017). As for the neuroimaging presentation, the thrombosis of cerebral sinus can be founded in the majority of patients with CVST (Anadure et al., 2018). Newly studies indicate that the regional variation of CVST can also influence the clinical presentation (Kalita et al., 2016; Wasay et al., 2019). For example, CVST patients in Asian countries have different clinical presentations, compared with in USA and other Western countries (Wasay et al., 2019). Therefore, it can be speculated that CVST patients at plateau and plain areas may have a variety of clinical differences as well. The alteration of hemostasis secondary to high‐altitude hypoxia‐induced polycythemia coupled with volume depletion and immobility can not only increase the risk of developing CVST but also can bring about characteristic clinical presentations (Zavanone et al., 2017). However, the existing literature on CVST at high altitude is very scant, and the accurate diagnosis is relatively difficult due to the wide spectrum of presenting symptoms (Zavanone et al., 2017). Correspondingly, it is imperative to clarify the clinical features and identify the presumed risk factors of CVST occurring at high altitude, which can contribute to the early diagnosis and treatment.

The purpose of this study is to compare the clinical differences of patients with CVST in plateau and plain areas and further ascertain the role of high‐altitude exposure in the etiology of aggravating predisposition toward CVST. Concretely, the clinical features, neuroimaging findings, medical history, hematological status, coagulation function, treatment methods, and final outcomes of CVST patients at high altitude and plain areas were documented in detail and comprehensively compared.

## 2 MATERIALS AND METHODS

### 2.1 Setting

This retrospective cross‐sectional study was conducted in China at The General Hospital of Western Theater Command in Chengdu and a tertiary hospital in plateau area. The clinical data of CVST patients in plain areas (altitude ≤ 1000 m) and plateau areas (altitude ≥ 4000 m) was collected from these two hospitals, respectively. Both two hospitals have independent neurovascular units for CVST patients and professional medical treatment teams consisting of neurovascular physician and nurse. Ethics Committee of the Faculty of The General Hospital of Western Theater Command in Chengdu and a tertiary hospital in plateau area gave permission for this research. All the studying procedures in this research were carried out in accordance with the approved guidelines.

### 2.2 Patients

CVST patients in plateau areas (altitude ≥ 4000 m) and plain areas (altitude ≤ 1000 m) were recruited from the inpatient service of a tertiary hospital in plateau area and The General Hospital of Western Theater Command in Chengdu, respectively, from June 2020 to December 2021. The diagnosis of CVST was made according to the clinical symptoms suggestive of CVST and contrast CT/MRV presentations (Bano et al., 2021): (1) presented with the clinical symptoms of CVST, including headache, focal neurological deficits, and cranial hypertension (diagnosed by the pressure detection of cerebrospinal fluid through lumbar puncture); (2) contrast cranial computed tomography (CT) scan showed a “delta sign”; (3) magnetic resonance imaging (MRI) or MR venography (MRV) demonstrated the cerebral sinus or venous occlusion (MRI and MRV were conducted by experienced radiologists who were not aware of the clinical symptoms and signs of patients to exclude the diagnosis deviation). The inclusion criteria include the following: (1) age ≥ 18 years; (2) discharge diagnosis of CVST (by using ICD 9 codes); (3) the time duration of patients with CVST in plateau areas (altitude ≥ 4000 m) was at least 1 months.; and (4) patients with CVST in plain areas (altitude ≤ 1000 m) had not exposed to high altitude during the past 2 years. The exclusion criteria include the following: (1) the admission and hospitalization information was incomplete; (2) the presence of extracranial thrombosis disease (such as venous thromboembolism/pulmonary embolism and so on); (3) preexisting a history or clinical symptoms suggestive of arterial stroke or primary intracerebral hemorrhage; and (4) combined with a paranasal sinus infection, cardiac/liver/renal/lung failure, hematological disease, malignancy, pregnancy, infection disease, and use of anticoagulants or antiplatelet agents.

### 2.3 Data collection

The clinical features, potential risk factors, neuroimaging findings, hematological status, treatment methods, and final outcomes of patients with CVST occurring at high altitude and plain areas were documented in detail and comprehensively analyzed by strict training researchers in our research team. Clinical features involved baseline demography, altitude and duration of plateau exposure, vital signs of hospital admission, time of onset, time before hospital admission, and key symptoms. Time of CVST onset was categorized as acute (less than 48 h), subacute (48 h to 30 days), and chronic (more than 30 days) according to the previous literature (Bousser, 2000). The key symptoms included isolated intracranial hypertension, headache, seizures, vomiting, focal weakness, visual impairment and diplopia, other neurological symptoms, and other presenting syndromes. Neuroimaging findings included the location and number of the thrombus, and the location and size of any parenchymal brain lesions. Hematological status mainly consisted of hematology profile, lipid profile, and coagulation profile. Specifically, the hematology profile included hemoglobin (HGB), red blood cells (RBCs) count, hematocrit (HCT), and total leukocyte count and total platelet (PLT) count. The lipid profile included total cholesterol (TC), triglyceride (TG), high‐density lipoprotein (HDL), and low‐density lipoprotein (LDL). The coagulation profile included prothrombin time (PT), thrombin time (TT), activated partial thromboplastin time (APTT), INR, d‐dimer (d‐D), and fibrinogen (FIB). Treatment methods included the anticoagulation drug such as unfractionated heparin or low‐molecular‐weight heparin (LMWH), and the endovascular treatment including local thrombolysis with infusion of the thrombolytic drug and mechanical thrombectomy with special devices. Final outcomes were evaluated by the neurological function and mRS score. According to mRS score, the outcome was classified into: complete recovery (mRS 0–1), partial recovery (independent mRS 2), dependent (mRS 3–5), and death (mRS 6).

### 2.4 Data Analysis

Measurement data was expressed as mean value ± standard deviations (M ± SD). Differences between two groups were analyzed by unpaired *t* test with Welch's correction. Relationships between two variables were evaluated using the Spearman rank correlation test. Enumeration data was analyzed by Chi‐squared test. SPSS version 18.0 software (SPSS Inc., USA) was used to perform the analysis, and two‐tailed *p* < .05 was considered statistically significant. Photoshop software (Adobe Software, Inc., USA) was used to draw the figure.

## 3 RESULTS

### 3.1 Patients selection

**FIGURE 1:**
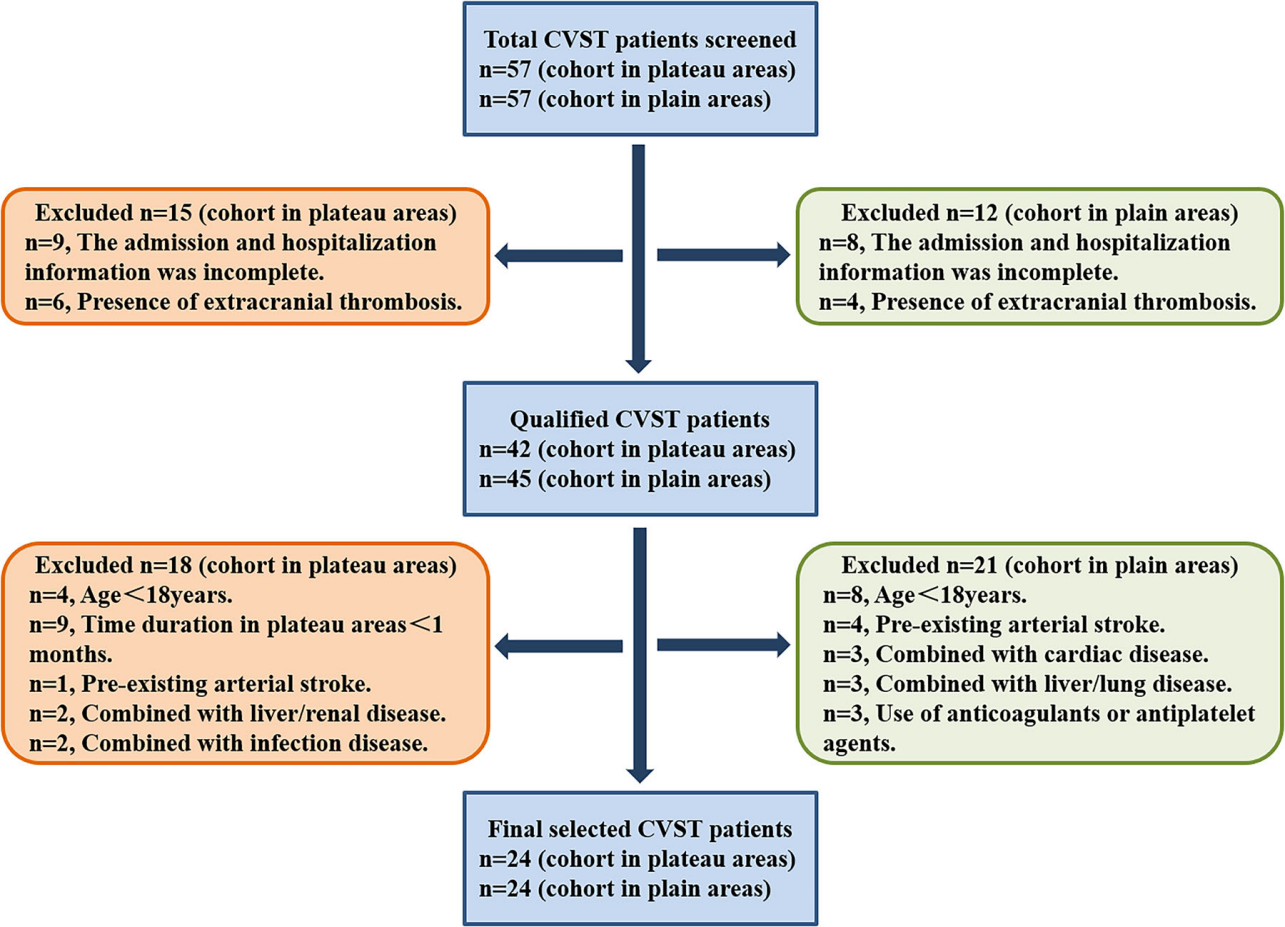
The selection flowchart of cerebral venous sinus thrombosis (CVST) patients in plateau and plain areas.

All CVST patients were chosen from the inpatient service of a tertiary hospital in plateau area and The General Hospital of Western Theater Command in Chengdu from June 2020 to December 2021 by using ICD‐9 procedural code terminology. Patients were qualified for recruitment if the diagnosis was isolated CVST, and they were further selected according to the inclusion and exclusion criteria as described in the “Materials and Methods” section. At last, 24 CVST patients in plateau areas (altitude ≥ 4000 m) and 24 CVST patients in plain areas (altitude ≤ 1000 m) were recruited. The selection flowchart is demonstrated in Figure 1.

### 3.2 Clinical features of CVST patients at plateau and plain areas

CVST patients at plateau and plain areas were parallel in the demographic baseline including gender, age, height, and weight, as well as the medical history of drinking and smoking. The mean altitude and time duration of CVST patients at plateau areas were 5200 ± 276.6 meters and 90.3 ± 107.6 days. CVST patients at plateau and plain areas were similar in the time before hospital admission, mean GCS score, onset type, and major symptoms. Moreover, the most common onset type of CVST occurring at plateau and plain areas was subacute (91.7% and 83.3% respectively), and the most frequent symptom was headache (37.5% and 33.3% respectively). As for the vital signs at hospital admission, CVST patients at plateau areas were analogical in body temperature, systolic blood pressure (SBP), diastolic blood pressure (DBP), and breathing frequency, but the heartbeat was slower in CVST patients at plateau areas (*p* < .001). Detailed data is showed in Table 1.

**TABLE 1:** Clinical features of cerebral venous sinus thrombosis (CVST) patients in plateau and plain areas

**Variable**	**CVST in plateau**	**CVST in plain**	***T*/*χ* ^2^ value**	***p* Value**
Gender (*n*)			n.a.	n.a.
Male	24	24		
Female	0	0		
Age (years)	27.5 ± 5.3	26.4 ± 6.2	0.676	.503
Height (cm)	173.0 ± 4.2	170.9 ± 4.8	1.593	.118
Weight (kg)	73.5 ± 7.7	70.8 ± 5.6	1.443	.156
Altitude of residence (m)	5200.0 ± 276.6	545.0 ± 58.1	80.681	<.001^*^
Duration at plateau (day)	90.3 ± 107.6	0	4.110	<.001^*^
Time before admission (h)	8.0 ± 4.9	11.4 ± 8.2	−1.757	.086
GCS	14.8 ± 0.5	14.3 ± 1.3	1.597	.117
Time of CVST onset (*n*, %)			0.762	.383
Acute	2 (8.3)	4 (16.7)		
Subacute	22 (91.7)	20 (83.3)		
Chronic	0 (0)	0 (0)		
Major symptoms (*n*, %)			1.268	.867
IIH	3 (12.5)	4 (16.7)		
Headache	9 (37.5)	8 (33.3)		
Seizures	4 (16.7)	2 (8.3)		
Vomiting	4 (16.7)	6 (25)		
Other	4 (16.6)	4 (16.7)		
Vital signs				
Body temperature (°C)	36.5 ± 0.35	36.7 ± 0.6	−1.121	.268
Heartbeat (times/min)	65.5 ± 13.3	83.8 ± 11.1	−5.172	<.001^*^
SBP (mmHg)	115.9 ± 15.5	121.6 ± 10.8	−1.496	.141
DBP (mmHg)	70.6 ± 9.3	75.1 ± 8.9	−1.707	.094
Respiratory rate	19.3 ± 1.7	19.6 ± 2.0	−0.614	.542
Medical history (*n*, %)				
With drinking history	11 (45.8)	9 (37.5)	0.343	.558
With smoking history	10 (41.7)	12 (50.0)	0.336	.562
Prothrombotic states	0	0		

*Note*: *p* < .05 represents statistically significant (marked with “*” and bold).

Abbreviations: DBP, diastolic blood pressure; GCS, Glasgow coma scale; IIH, isolated intracranial hypertension; SBP, systolic blood pressure.

### 3.3 CVST patients at plateau and plain areas had similar neuroimaging findings

CVST patients at plateau and plain areas were diagnosed with similar neuroimaging techniques, including cranial CT, MRI, and MRV, of which the most frequent one was cranial MRI + MRV (54.2% and 45.8%, respectively). Neuroimaging findings showed that the most common sinus affected was sigmoid sinus (16 and 14, respectively), followed by superior sagittal sinus and right transverse sinus, and the distribution of sinus involvement had no significant difference between CVST patients at two areas. In addition, the most common number of sinus involvement was “more than two” (66.7% and 58.3%, respectively), the number of sinus involvement and the MRI presentation (hemorrhage/infarcts) had no significant difference between two groups. These results indicated that CVST patients at two areas had similar neuroimaging findings. Detailed data is showed in Table 2.

**TABLE 2:** Neuroimaging findings of cerebral venous sinus thrombosis (CVST) patients in plateau and plain areas

**Variable (*n*, %)**	**CVST in plateau**	**CVST in plain**	***χ* ^2^ value**	***p* Value**
**Neuroimaging workup**			0.389	.943
Cranial MRI	4 (16.7)	5 (20.8)		
Cranial MRV	3 (12.5)	3 (12.5)		
Cranial MRI+MRV	13 (54.2)	11 (45.8)		
Cranial CT+MRV	4 (16.7)	5 (20.8)		
**Name of sinuses involved**			2.82	.588
Superior sagittal sinus	12	10		
Left transverse sinus	6	7		
Right transverse sinus	12	8		
Sigmoid sinus	16	14		
Straight sinus	5	10		
**Number of sinuses involved**			0.367	.832
One sinus	3 (12.5)	4 (16.7)		
Two sinuses	5 (20.8)	6 (25)		
More than two sinuses	16 (66.7)	14 (58.3)		
**Cerebral MRI presentation**			0.873	.646
CVST only	15 (62.5)	18 (75)		
Cerebral hemorrhage	6 (25)	4 (16.7)		
Cerebral infarction	3 (12.5)	2 (8.3)		

*Note*: *p* < .05 represents statistically significant (marked with * and bold).

Abbreviations: HCT, hematocrit; HDL high‐density lipoprotein; HGB, hemoglobin; PLT, platelet; RBCs, red blood cells.

### 3.4 CVST patients at plateau areas presented with altered coagulative state

As shown in Table 3, the HGB level, RBCs count, and HCT were obviously higher (both *p* < .001) in CVST patients at plateau areas than at plain areas, as well as the total leukocyte count (*p* = .016). In addition, the lipid profile analysis indicated that CVST patients at plateau areas had lower level of HDL (*p* < .001) but had similar level of TC, TG, and LDC with CVST patients at plain areas. In the analysis of coagulation function, CVST patients at plateau areas had considerably longer PT, APTT, TT, and INR (*p* = .017, .010, .015, and .001, respectively), and obviously higher level of d‐D (*p* < .001) than CVST patients at plain areas. These results indicated that CVST patients at plateau areas presented with altered coagulative state.

**TABLE 3:** Hematology/lipid/coagulation profile of cerebral venous sinus thrombosis (CVST) patients in plateau and plain areas

**Variable**	**CVST in plateau**	**CVST in plain**	***T* Value**	***p* Value**
**Hematology**				
HGB (mg/dL)	176.5 ± 25.8	143.0 ± 18.5	5.169	<.001^*^
RBCs (10^12^/L)	5.9 ± 0.6	5.0 ± 1.0	4.018	<.001^*^
HCT (%)	50.9 ± 6.8	42.1 ± 5.5	4.968	<.001^*^
PLT (10^9^/L)	211.3 ± 15.1	222.2 ± 83.8	−0.628	.533
Total leukocytes (10^9^/L)	11.5 ± 5.0	8.7 ± 2.4	2.505	.016^*^
**Lipid profile (mg/dL)**				
TC (mmol/L)	3.9 ± 0.9	4.3 ± 1.3	−1.249	.218
TG (mmol/L)	1.3 ± 0.5	1.5 ± 0.5	−1.318	.194
HDL (mmol/L)	0.9 ± 0.2	1.3 ± 0.2	−6.378	<.001^*^
LDL (mmol/L)	2.4 ± 0.6	2.6 ± 1.0	−0.931	.357
**Coagulation profile**				
PT (s)	15.1 ± 7.1	11.4 ± 1.9	2.474	.017^*^
APTT (s)	33.2 ± 8.1	26.8 ± 8.4	2.671	.010^*^
TT (s)	20.9 ± 4.7	18.1 ± 2.8	2.527	.015^*^
INR	1.28 ± 0.6	0.97 ± 0.1	2.461	.001^*^
FIB (mg/dL)	3.1 ± 1.1	3.1 ± 1.2	0.054	.957
d‐D (mg/L)	6.5 ± 4.5	1.3 ± 0.9	5.633	<.001^*^

*Note*: *p* < .05 represents statistically significant (marked with * and bold).

Abbreviations: APTT, activated partial thromboplastin time; DD, d‐dimer; FIB, fibrinogen; HCT, hematocrit; HDL, high‐density lipoprotein; HGB, hemoglobin; INR, international normalized ratio; LDL, lower‐density lipoprotein; PLT, platelet; PT, prothrombin time; RBCs, red blood cells; TC total cholesterol; TG, triglycerides; TT, thrombin time.

### 3.5 Correlation between basic coagulation parameters and RBCs indexes in CVST patients

In order to explore the coagulative state of CVST patients furthermore, the correlations between basic coagulation parameters (APTT, PT, TT, FIB, and PLT) and RBCs indexes (HGB and HCT) were analyzed. As demonstrated in Table 4, CVST patients at plateau areas had positive correlation between PT and HGB/HCT (*r* = .610 *p* = .002/*r* = .530 *p* = .008), APTT and HGB/HCT (*r* = .646 *p* = .001/*r* = .523 *p* = .009), FIB and HGB/HCT (*r* = .825 *p* < .001/*r* = .594 *p* = .002), and PLT and HCT (*r* = .488 *p* = .015). In addition, CVST patients at plain areas had positive correlation between PT and HCT (*r* = .430 *p* = .036), APTT and HCT (*r* = .417 *p* = .042) but had negative correlation between PLT and HGB/HCT (*r* = −.506 *p* = .012/*r* = −.485 *p* = .016). Next, the correlation between basic coagulation parameters APTT, PT, TT, and PLT, d‐D and FIB were analyzed as well. As shown in Table 5, FIB was positively correlated with d‐D in CVST patients at plain areas (*r* = .428, *p* = .037), but they had no correlation at plateau areas. In addition, no correlation was found between APTT, PT, and TT and PLT in CVST patients at plateau and plain areas.

**TABLE 4:** Correlation between coagulation parameters and red blood cells (RBCs) indexes of cerebral venous sinus thrombosis (CVST) patients

	**CVST in plateau areas**		**CVST in plain areas**	
	HGB (mg/dL) (*r*, *p*)	HCT(%) (*r*, *p*)	HGB (mg/dL) (*r*, *p*)	HCT(%) (*r*, *p*)
PT (s)	.610, .002^*^	.530, .008^*^	.338, .107	.430, .036^*^
APTT (s)	.646, .001^*^	.523, .009^*^	.367, .078	.417, .042^*^
TT (s)	.344, .099	.356, .087	−.125, .562	−.134, .532
FIB (mg/dL)	.825, <.001^*^	.594, .002^*^	.088, .683	.111, .605
PLT (10^9^/L)	.400, .053	.488, .015^*^	−.506, .012^*^	−.485, .016^*^

*Note*: *p* < .05 represents statistically significant (marked with * and bold).

Abbreviations: APTT, activated partial thromboplastin time; FIB, fibrinogen; HCT, hematocrit; HGB, hemoglobin; PLT, platelet; PT, prothrombin time; TT, thrombin time.

**TABLE 5:** Correlation between coagulation parameters of cerebral venous sinus thrombosis (CVST) patients

	**CVST in plateau areas**		**CVST in plain areas**	
	PLT (10^9^/L) (*r*, *p*)	DD (mg/L) (*r*, *p*)	PLT (10^9^/L) (*r*, *p*)	DD (mg/L) (*r*, *p*)
PT (s)	.055, .800	n.a.	.012, .957	n.a.
APTT (s)	.263, .214	n.a.	−.041, .848	n.a.
TT (s)	.231, .278	n.a.	.280, .185	n.a.
FIB (mg/dL)	n.a.	−.301, .152	n.a.	.428, .037^*^

*Note*: *p* < .05 represents statistically significant (marked with * and bold).

Abbreviations: APTT, activated partial thromboplastin time; DD, d‐dimer; FIB, fibrinogen; PLT, platelet; PT, prothrombin time; TT, thrombin time.

### 3.6 CVST patients at plateau areas and plain areas had similar treatment and outcome

At last, the differences of treatment and outcome in CVST patients at two areas were analyzed. As shown in Table 6, the anticoagulation drug LMWH was used in most of CVST patients at plateau and plain areas (83.3% vs. 87.5%, *p* = .683). However, the endovascular therapy was performed in only a small part of CVST patients at plateau and plain areas (20.8% vs. 37.5%, *p* = .434). In addition, the rate of endovascular therapy application was higher in CVST patients at plain areas than at plateau areas, but without significant difference. In addition, CVST patients at plateau areas gained similar recovery outcome with CVST patients at plain areas.

**TABLE 6:** Treatment and outcome of cerebral venous sinus thrombosis (CVST) patients in plateau and plain areas

**Variable**	**CVST in plateau**	**CVST in plain**	***T*/*χ* ^2^ Value**	***p* Value**
**Anticoagulation drug (*n*, %)**			0.167	.683
Unfractionated heparin	4 (16.7)	3 (12.5)		
LMWH	20 (83.3)	21 (87.5)		
**Endovascular therapy (*n*, %)**			1.671	.434
Local thrombolysis	2 (8.3)	3 (12.5)		
Mechanical thrombectomy	3 (12.5)	6 (25)		
None	19 (79.2)	15 (62.5)		
**mRS classification (*n*, %)**			2.100	.552
Complete recovery	14 (58.3)	18 (75)		
Partial recovery	6 (25)	4 (16.7)		
Dependent	3 (12.5)	2 (8.3)		
Death	1 (4.2)	0 (0)		

*Note*: *p* < .05 represents statistically significant (marked with * and bold).

Abbreviations: LMWH, low‐molecular‐weight heparin; mRS, modified Rankin scale score.

## 4 DISCUSSION

To our knowledge, this is the first study that investigated the clinical differences of patients with CVST in plateau and plain areas. Main findings were as follows: (1) CVST patients in plateau areas had longer time before hospital admission and slower heartbeat than CVST patients in plain areas; (2) the most common onset type of CVST occurring at plateau and plain areas was subacute, and the most frequent symptom was headache; (3) neuroimaging findings of CVST patients in plateau and plain areas were similar with each other; (4) CVST patients in plateau areas presented with altered coagulative state; (5) CVST patients in plateau areas had received similar treatment and gained similar recovery outcome with CVST patients in plain areas. These findings are of great significance to clarify the clinical features of CVST patients in plateau areas.

CVST is a rare but significant cause of stroke, which is associated with various risk factors, including inherited thrombophilia, acquired prothrombotic state, oral contraceptives, and so on (Appenzeller et al., 2005). Latest studies indicate that high‐altitude exposure might be a risk factor for CVST as well, as the risk of venous thrombotic events is reported to increase by 30 times at high altitude (Anand et al., 2001; Devasagayam et al., 2016; Hassan et al., 2019; Khattar et al., 2019). Nevertheless, the exact prevalence and mechanism of CVST at high altitudes are still unknown, probably because the literature of CVST at high altitude is scarce and contains only a few case reports or case series. According to the existed literature, there might be several factors contributing to the occurrence of CVST at high altitude. First, the slowing of cerebral blood flow may play an important pathogenetic role, as high‐altitude exposure leads to the increase of blood viscosity that further causes the decrease of cerebral blood flow and oxygen transport (Song et al., 1986). Second, preexisting prothrombotic states, such as hereditary thrombophilia, may be the most likely predisposing factors for CVST occurring at high altitude (Zavanone et al., 2017). Moreover, other factors, including secondary polycythemia, hypoxia‐induced hemostatic alterations such as increased platelet activity, inflammatory changes secondary to endothelial injury, and coagulation pathway activation triggered by hypothermia might play essential roles in the occurrence of CVST at high altitude as well (Zavanone et al., 2017). These issues would be explored if much more studies with a large sample could be conducted in the future.

CVST is more frequent in female than in male at plain areas, as previous studies indicated that the incidence of CVST in women was threefold higher due to the use of oral contraceptives and puerperium (Coutinho et al., 2009; Ferro et al., 2004). However, the majority of CVST patients at plateau areas were males, and females developing CVST at plateau areas are limited (Cheng et al., 2009). Similarly, all the CVST patients at plateau areas in this study were males. The reason for men are more susceptible to CVST at high altitude is unclear; one probable explanation is that most of the research subjects are men who usually work at plateau areas (Song et al., 1986; Torgovicky et al., 2005). CVST patients usually present with a wide range of clinical symptoms, including headache, seizures, vomiting, altered consciousness, and papilledema, of which the most common one is headache (Coutinho, 2015; Coutinho et al., 2015). This conclusion is consistent with our study, as 37.5% and 33.3% CVST patients at plateau and plain areas present as headache. Headache usually reflects the increase of intracranial pressure, which is also a common symptom of other neurological conditions occurring at high altitude such as acute mountain sickness or high‐altitude cerebral edema. Herein, the diagnosis of CVST at plateau areas might face some challenges, as the symptom is varying and misleading. In order to improve the accuracy, neuroimaging techniques, such as CT, CTV, MRI, and MRV, have been generally applied in the accurate diagnosis of CVST. Noninvasive imaging significantly increases the sensitivity of diagnosis, as MRV is the most sensitive diagnostic modality and CTV is a reasonable alternative to MRV for the diagnosis of CVST, both of which have equivalent sensitivity and specificity (Ferro et al., 2017; Lafitte et al., 1997; Silvis et al., 2017). In our study, the most frequently used neuroimaging techniques were cranial MRI+MRV in CVST patients at plateau and plain areas. In addition, neuroimaging findings indicated that the most common sinus affected was sigmoid sinus, the most common number of sinus involvement was “more than two”, without difference between two groups. Our results might be inconsistent with other studies, because the proportion of sinus involvement was quite variable due to the type and timing of neuroimaging (Devasagayam et al., 2016; Kalita et al., 2016; Wang et al., 2019; Zhou et al., 2017).

Coagulative function plays an important role in the mechanism of CVST, especially in the occurrence of CVST at high altitude. Our study showed that CVST patients at plateau areas presented with altered coagulative state. First, CVST patients at plateau areas presented with higher level of HGB, RBCs, HCT, and d‐D, in comparison with CVST patients at plain areas. RBCs play an essential role in the adaptation to hypoxia at plateau areas due to their vital function in the process of systemic oxygen transportation and delivery (D'Alessandro et al., 2016). High‐altitude leads to the elevation of HGB and HCT levels, which promotes the oxygen‐carrying capacity of RBCs but aggravates the risk of thrombosis (Li et al., 2018; Liu et al., 2017). High altitude brings about hypercoagulability manifested as increased d‐D levels, which are usually applicated in the diagnosis of venous thrombotic diseases, but cannot be reliably used to exclude CVST due to their high negative predictive value (Dentali et al., 2012; Hiltunen et al., 2013). As the d‐D level was obviously higher in CVST patients at plateau areas than at plain areas, d‐D testing might be useful to make a diagnosis for patients with the suspicion of CVST at plateau areas. Second, PT, APTT, TT, and INR were found to be obviously higher in CVST patients at high altitude, which was consistent with the result of other studies (Damodar et al., 2018; Vij, 2009; Zhang et al., 2019) but was contradictory to the conclusion that high‐altitude activated the coagulation cascade and caused the increased risk of thrombosis (Damodar et al., 2018). These results can be explained by the influence of high‐altitude exposure, which induced the compensatory hyperplasia of RBCs and the elevation of HCT and further led to the prolongation of PT, APTT, and TT, as our and other studies both demonstrated that HCT was positively correlated with PT, APTT (Marlar et al., 2006; Zhang et al., 2019). Third, CVST patients at plateau areas had positive correlation between PT and HGB/HCT, APTT and HGB/HCT, FIB and HGB/HCT, and PLT and HCT. In addition, CVST patients at plain areas had positive correlation between PT and HCT, APTT and HCT but had negative correlation between PLT and HGB/HCT. In addition, FIB was positively correlated with d‐D in CVST patients at plain areas, but they had no correlation at plateau areas. It is worth noting that the correlations between these variables were weak, which might have little clinical significance. Moreover, no studies have studied the coagulation function of CVST patients at plateau areas so far. Herein, it might be difficult to make precise conclusions based on these results, and further studies will be needed to analyze the influence of high altitude on the coagulation function in CVST patients.

The alternative treatments for CVST include anticoagulation drugs such as unfractionated heparin and LMWH, and endovascular operations such as local thrombolysis with the thrombolytic drug infusion and mechanical thrombectomy with special devices (Saposnik et al., 2011; Stam et al., 2008). At present, no convincing studies have evaluated the efficacy and safety of endovascular treatment for CVST, except several case reports mentioned the therapeutic effect of local thrombolysis in CVST (Guo et al., 2012; Li et al., 2013; Stam et al., 2008). Our study showed that the anticoagulation drug LMWH was used in most of CVST patients at plateau and plain areas (83.3% vs. 87.5%), but the endovascular therapy was performed in only a small part of CVST patients at plateau and plain areas (20.8% vs. 37.5%). As for the final outcome, CVST patients at plateau areas gained similar recovery outcome with CVST patients at plain areas. These results indicated that CVST patients in plateau areas had received similar treatment protocols and gained similar recovery outcome with CVST patients in plain areas.

There are several limitations in this study. First, this study is an observational and retrospective one without intervention and follow‐up visits. Second, the number of subjects is relatively small. Third, the gender of recruited patients is only male because of the particular geographical environment in plateau areas restricted the population recruitment. At last, the outcome of this study is relatively underpowered, and much more studies with large sample will be needed in the future.

In conclusion, this retrospective cross‐sectional study demonstrated that CVST patients in plateau areas presented with longer time before hospital admission, altered coagulation function, and aggravated predisposition toward venous thromboembolism compared with CVST patients in plain areas. The most common symptom was headache, the most common sinus affected was sigmoid sinus, and the most common number of sinus involvement was “more than two,” without differences between CVST patients in plateau and plain areas. In addition, CVST patients in plateau areas and plain areas received similar treatment protocols and gained similar recovery outcome. These findings are of great significance to clarify the clinical features of CVST patients in plateau areas.

## AUTHOR CONTRIBUTIONS

Yuan Ma and Yongjian Yang designed the study and guided the writing of this article. Yongxiang Yang, Jingmin Cheng, and Yuping Peng were responsible for conducting the study and writing the manuscript. Yan Luo and Dongbo Zou contributed to acquire and analyze the data. Authors included in this article agreed with the final manuscript.

## CONFLICT OF INTEREST STATEMENT

The authors declare no conflict of interest.

### PEER REVIEW

The peer review history for this article is available at https://publons.com/publon/10.1002/brb3.2998.

## Data Availability

The data that supports the findings of this study is available from the corresponding author upon reasonable request.
